# Diversity of soil faunal community as influenced by crop straw combined with different synthetic fertilizers in upland purple soil

**DOI:** 10.1038/s41598-022-23883-6

**Published:** 2022-11-11

**Authors:** Xiuhong Xie, Xuefeng Wang, Zhixin Dong, Bo Zhu

**Affiliations:** 1 Key Laboratory of Mountain Surface Processes and Ecological Regulation, Institute of Mountain Hazards and Environment, Chinese Academy of Sciences, Chengdu, 610041 China; 2 Institute of Mountain Hazards and Environment, Chinese Academy of Sciences, No. 9, Section 4, South Renmin Road, Chengdu, 610041 Sichuan China; 3 University of Chinese Academy of Sciences, Beijing, 100049 China; 4 Jilin Agricultural University, Changchun, 130118 China

**Keywords:** Ecology, Biodiversity

## Abstract

Soil fauna play a crucial role in sustaining agro-ecosystem functions. Crop straw is recommended for application to agricultural fields to improve soil quality. However, the effects of crop straw combined with different synthetic fertilizers on the soil faunal community remain unclear, and knowledge regarding purple soil is limited. Using the conserved cytochrome c oxidase I (COI) gene as markers, we examined the responses of the soil faunal community to different fertilization in upland purple soil of southwestern China. The accuracy of the morphological and molecular methods in characterizing soil nematodes was compared. Our results showed that different fertilization treatments significantly changed the soil faunal community structure (Adonis test, *R*^2^ = 0.43, *P* = 0.011). Sixteen biomarkers were identified according to LEfSe (linear discriminant analysis effect size). The diversity and species number of soil fauna were closely related to soil organic matter (SOM) and total phosphorus (TP) (*P* < 0.05). This study indicates that crop straw return can improve the soil fertility and diversity of soil fauna in purple soil. Additionally, the morphological approach and molecular method based on the COI gene can be considered as complementary approaches in characterizing soil nematode community.

## Introduction

Soil fauna are an important part of the food webs in the soil ecosystem and play multiple roles as consumers and decomposers^1^. They directly or indirectly take part in sustaining ecosystem functions, including litter decomposition, soil pollution abatement, plant community dynamics, maintenance of soil structure, and carbon, nitrogen and phosphorus cycling^2–5^. Soil fauna are highly diverse, which are generally divided into microfauna, mesofauna, macrofauna and megafauna based on different body widths^6^. They are sensitive to changes in their habitat^7^, such as fertilizer use, tillage, and multiple species, i.e., earthworms and oribatids can be used as biological indicators to indicate changes in soil fertility and quality^8^.

Crop straw return to agricultural fields is an important measure to improve soil fertility and increase yield^9–11^, since the straw contains necessary ingredients for plants to grow, including calcium, magnesium, nitrogen, phosphorus, potassium and organic matter^12^. Crop straw return affects soil physical and chemical properties^10^, which may further change the community and diversity of soil fauna^13^. For example, crop residues increased soil microarthropod abundance but had no significant effect on biodiversity in loamy sand^14^; another study on soil meso- and microarthropods in tidal soil reported that straw returned to wheat–maize fields reduced the number of soil faunal groups^15^. However, in another study, the return of crop residues with synthetic fertilizer, nitrogen, phosphorus and potassium, caused a significant decrease in the number of nematodes and an increase in the number of earthworms and ground beetles in sod-podzolic gley mid-loamy soil^7^. Straw return provided richer living conditions, and the dominant groups, including Oribatida and Actinedida, accounted for 77.1% of the meso- and microfauna population in black soil^16^, while the dominant groups in fluvo-aquic soils were Collembola and Acari, which accounted for nearly 80%^17^. Through bottom-up and top-down processes, organic fertilizers, including straw residues, have a strong impact on a wide range of biological communities and enhance the processes of decomposition and carbon, nitrogen and phosphorus cycling in the soil food webs^18,19^. Therefore, fertilizer application generally has positive, negative, or no significant effects on soil faunal abundance (earthworms, Collembola, Acari, and nematodes, respectively) and diversity^19^. The impact of straw return on soil fauna remains inconsistent because of different soil types, land use conditions and chemical fertilizer amendments. However, few investigations have focused on overall micro, meso- and macrofauna^20^, and there remains much uncertainty regarding the impact of straw return plus different chemical fertilizers on their abundance and diversity.

Purple soil is the most important soil type in southwestern China and is mainly distributed in the hilly area of the Sichuan Basin and the low mountain area below 800 m above sea level^21^. Developing from calcareous purple sedimentary parent material, this mineral-rich purple soil combines with the tropical monsoon climate to make the region the most important agricultural zone in Southwest China with extensive cultivation and production of rich agricultural products^22^. Nevertheless, purple soil lacks organic matter and phosphorus due to extensive soil erosion and degradation^23^; straw return is recommended for application to agricultural fields to improve soil quality and fertility^10,24^. However, knowledge of the response of integral soil fauna in upland purple soil to straw return plus chemical fertilizers is limited^25^.

Traditionally, studies on the soil faunal community and diversity have mostly used taxonomy based on morphology as the identification species method, but this approach is time-consuming and laborious^26^. Moreover, because of the fuzziness and concealment of morphological approaches in characterizing soil fauna, their diversity is difficult to describe^27^. The cytochrome c oxidase subunit I (COI) mitochondrial DNA (mtDNA) marker is an official animal barcode marker. And COI metabarcoding technique is developing rapidly which has been frequently used to investigate the soil faunal community and diversity^28–30^. For example, McGee et al.^31^ and Porter et al.^32^ studied soil fauna in tropical secondary forests and terrestrial arthropods, respectively, and concluded that COI coding of soil arthropods was an effective biological monitoring method. Morise et al.^33^ explored the nematode community in nonmanaged flower soil and indicated that the COI gene technique provided a detailed structure (23 COI gene-derived operational taxonomic units, or OTUs) of the nematode community. Drummond et al.^34^ extracted soil environmental DNA (eDNA) and found that the COI genetic method and traditional morphology had a high correlation and believed that the COI genetic marker was the best alternative marker for soil biodiversity. Dopheide et al.^35^ contrasted 28S rRNA with the COI technique to estimate the biodiversity of terrestrial fauna on forest islands, suggesting that the COI genetic technique was more reliable than the 28S rRNA genetic technique for species richness estimation and identification.

These studies showed that the COI gene coding technique is effective in characterizing the soil faunal community. However, it had been reported that this approach remained limited. Watts et al.^36^ and Kvist^37^ stated that the current COI metabarcoding method was not ideal because the COI sequence databases (NCBI and BOLD) were devoid of data for many animal groups, and few OTUs could be confidently identified to the genus level. As stated above, the COI gene coding technique needs to be validated in determining the taxonomic identity of the soil faunal community. Soil nematodes are one of the most numerous of all multicellular organisms, occupying many key nodes of the soil food webs^38,39^. Here, we simultaneously observed the diversity and composition of soil nematodes using a traditional morphological method and compared it with a molecular method based on the COI gene.

Therefore, the main objectives of this study were to explore (1) the differences in the identification of soil nematodes between the COI genetic technique and traditional morphological method; (2) the effects of straw return combined with different synthetic fertilizers on the composition and diversity of the purple soil faunal community; and (3) the relationship between soil faunal abundance, diversity and soil properties under different fertilization treatments.

## Results

### The impact of different fertilization on soil properties

Different fertilization regimes significantly influenced the soil AP, NO_3_^–^N, TDN, TP, TN, SOM and WC (Table 1). The SOM and TN of the purple soil under the straw return treatments (RSDNP, RSDN and RSDNPK) were significantly higher than those in the absence of straw return (NPK and N) (*P* < 0.05). Additionally, the soil TDN, NO_3_^–^N and WC in RSDNP, RSDN and RSDNPK were significantly higher than those under N fertilization alone (*P* < 0.05). The application of phosphate fertilizer (NPK, RSDNP and RSDNPK) improved soil TP compared with the treatment without phosphate (N and RSDN). The effects of different fertilization regimes on the contents of soil pH, TK, DOC, NH_4_^+^-N and AK were not significant (*P* > 0.05). Purple soil is rich in potassium^23^, and there was no significant difference in the soil characteristics between the RSDNP and RSDNPK fertilization treatments.

**Table 1:** Soil properties under different fertilizer regimes.

Soil properties	N	NPK	RSDN	RSDNP	RSDNPK
WC	18.15 ± 0.31b	19.67 ± 0.51a	22.21 ± 0.24a	22.59 ± 1.52a	22.30 ± 0.74a
pH	8.33 ± 0.09a	8.24 ± 0.09a	8.30 ± 0.09a	8.18 ± 0.09a	8.20 ± 0.09a
SOM (g kg^−1^)	8.12 ± 0.17b	9.91 ± 0.50b	14.51 ± 0.89a	16.46 ± 1.20a	16.52 ± 1.48a
TN (g kg^−1^)	0.68 ± 0.32c	0.86 ± 0.28b	1.15 ± 0.54a	1.26 ± 0.83a	1.20 ± 0.60a
TP (g kg^−1^)	0.71 ± 0.03b	1.11 ± 0.09a	0.68 ± 0.05b	1.30 ± 0.10a	1.10 ± 0.18a
TK (g kg^−1^)	20.02 ± 0.67a	18.94 ± 0.82a	20.21 ± 1.69a	19.84 ± 1.03a	20.61 ± 0.98a
TDN (mg kg^−1^)	10.97 ± 0.88b	16.63 ± 3.01a	24.02 ± 1.70a	23.18 ± 2.31a	25.30 ± 7.48a
NO_3_^–^N (mg kg^−1^)	2.33 ± 0.64b	4.68 ± 2.16ab	7.98 ± 1.51a	7.38 ± 1.62a	6.34 ± 3.38a
NH_4_^+^-N (mg kg^−1^)	0.65 ± 0.16a	0.82 ± 0.04a	1.19 ± 0.03a	1.22 ± 0.11a	3.32 ± 2.26a
AP (mg kg^−1^)	3.30 ± 0.1c	20.79 ± 1.46ab	5.21 ± 0.28b	31.14 ± 6.13a	11.48 ± 5.59ab
AK (mg kg^−1^)	100.98 ± 2.08a	121.20 ± 4.03a	166.36 ± 15.25a	139.51 ± 8.98a	170.09 ± 39.71a
DOC (mg kg^−1^)	44.67 ± 1.50a	47.13 ± 1.99a	66.98 ± 4.09a	51.82 ± 14.43a	63.93 ± 4.82a

*SOM* soil organic matter, *TN* total nitrogen, *TP* total phosphorus, *TK* total potassium, *AP* available phosphorus, *AK* available potassium, *TDN* dissolved total nitrogen, *DOC* dissolved organic carbon, *NH*_*4*_^*+*^*-N* ammonium nitrogen, *NO*_*3*_^*–*^*N* nitrate nitrogen, *WC* water content, *N* synthetic N fertilizer, *NPK* synthetic fertilizer: nitrogen, phosphorus and potassium, *RSDN* crop residues returned with nitrogen, *RSDNP* crop residues returned with nitrogen and phosphorus, *RSDNPK* crop residues returned with nitrogen, phosphorus and potassium.

Within a row, values (mean ± standard error) with different letters (a, b and c) indicate significant differences (one-way ANOVA with LSD test, *P* < 0.05; n = 3) among the fertilization treatments.

### The soil nematode community identified by traditional morphological and molecular biological methods

In this study, both traditional morphological and molecular biological methods were used to identify soil nematodes. A total of 508 nematodes were isolated by the traditional morphological method and belonged to three classes, eight orders, twenty families and thirty-eight genera (Supplementary Table S1). At the genus level, *Acrobeloides* and *Mesorhabditis* were the dominant groups (relative abundance > 5%), and the common groups (5% > relative abundance > 1%) were *Epidorylaimus* and *Plectus.*

Based on the mitochondrial COI gene (Supplementary Table S2), we observed that 4225 OTUs of the nematode community belonged to two classes, three orders, seven families and nine genera. *Cylicostephanus* was the most dominant genus, and *Schistonchus*, *Fergusobia*, *Xiphinema*, and *Thelandros* were the common genera. Some parasitic nematodes (*Heterakidae, Ancylostomatidae*) were also detected by the molecular biological method.

### Community composition of soil fauna

Based on a COI gene sequence similarity cutoff value of 97%, 357,634–463,460 OTUs were observed in the 15 soil samples. Soil fauna were classified by species annotation and belonged to 16–18 phyla, 37–42 classes, 111–125 orders, 315–349 families and 290–509 genera across all samples.

**Figure 1:**
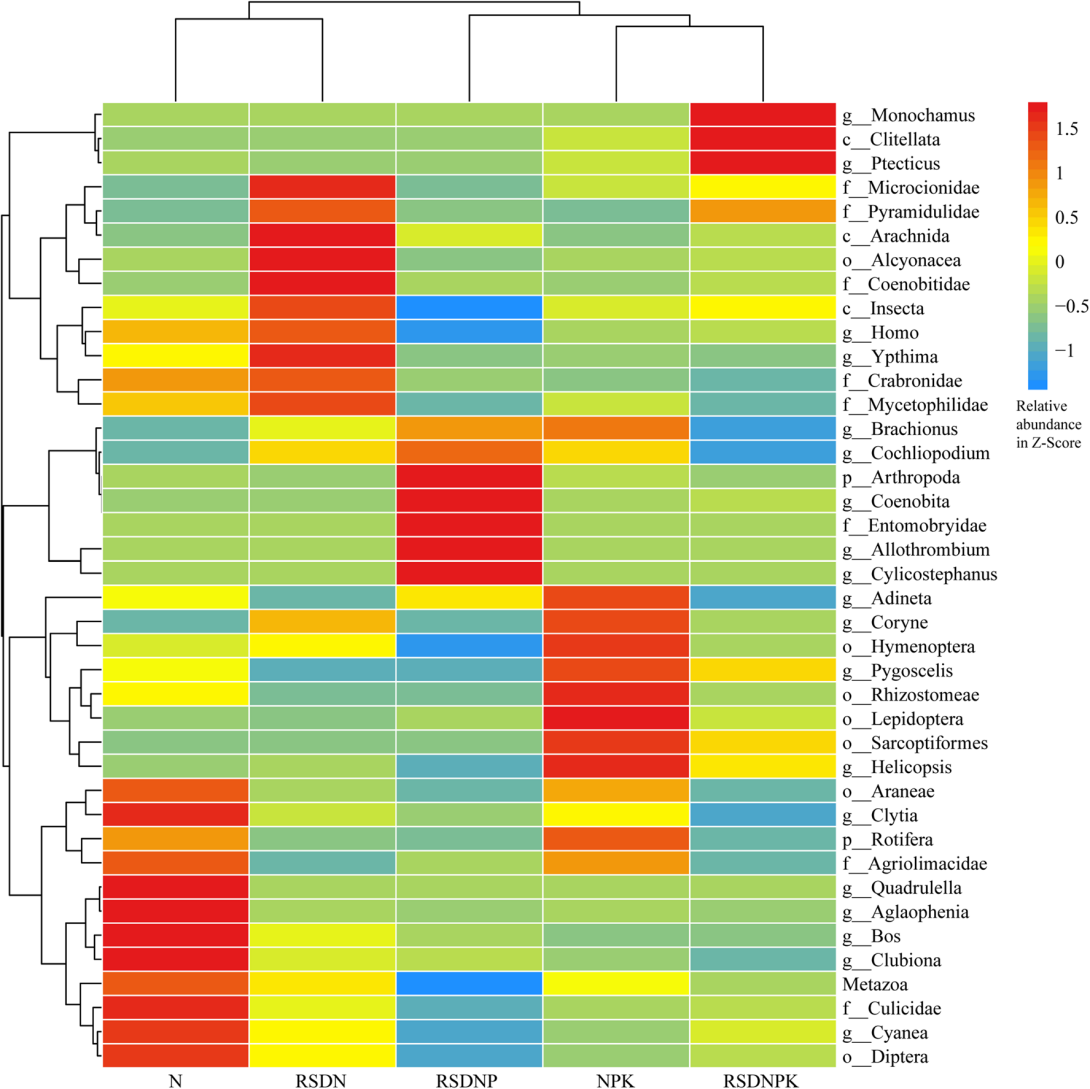
Heatmap of the top 40 abundant soil faunal taxa based on genus level under different fertilization regimes. *N* synthetic N fertilizer, *NPK* synthetic fertilizer: nitrogen, phosphorus and potassium, *RSDN* crop residues returned with nitrogen, *RSDNP* crop residues returned with nitrogen and phosphorus, *RSDNPK* crop residues returned with nitrogen, phosphorus and potassium. When the genus was unclassified, the corresponding higher-level annotation was adopted and denoted by f_—_, o_—_, c_—_, or p_—_.

The difference in the total soil faunal community among the fertilization treatments was significant (Adonis test, *R*^2^ = 0.43, *P* = 0.011). The heatmap (Fig. 1) showed the top 40 abundant soil faunal taxa. Cluster analysis (Fig. 1) showed that the RSDNP, RSDNPK, and NPK treatments and the RSDN and N treatments were clustered into two categories (without phosphate fertilizer: RSDN, N and with phosphate fertilizer: RSDNP, RSDNPK, NPK), which indicated that the application of phosphate fertilizer significantly changed the composition of the soil faunal community.

The dominant taxa in the N treatment were Araneae (5.34%) and Insecta (8.42%), and the common taxa were *Quadrulella* (4.2%), Rotifera (3.03%) and Lepidoptera (2.80%). The highest abundance of taxa in the RSDN treatment was detected in Insecta (13.31%), followed by *Coryne* (6.55%), Alcyonacea (6.27%), *Ypthima* (3.54%), Microcionidae (2.75%), Lepidoptera (2.65%), Araneae (1.76%) and Arachnida (1.43%).

The dominant taxa in the NPK treatment were *Coryne* (10.57%), Insecta (8.12%), and Lepidoptera (7.84%), and the common taxa were Araneae (4.10%) and Rotifera (3.80%). In the RSDNPK treatment, the dominant taxa were Clitellata (8.99%), *Ptecticus* (17.29%), and Insecta (9.16%), and the common taxa were *Monochamus* (4.12%) and Lepidoptera (3.47%). In addition, the highest abundance of taxa in RSDNP was Entomobryidae (11.03%), and Insecta (3.54%), *Coenobita* (3.16%), Lepidoptera (3.13%) and *Allothrombium* (2.95%) were the common taxa in the RSDNP treatment.

**Figure 2:**
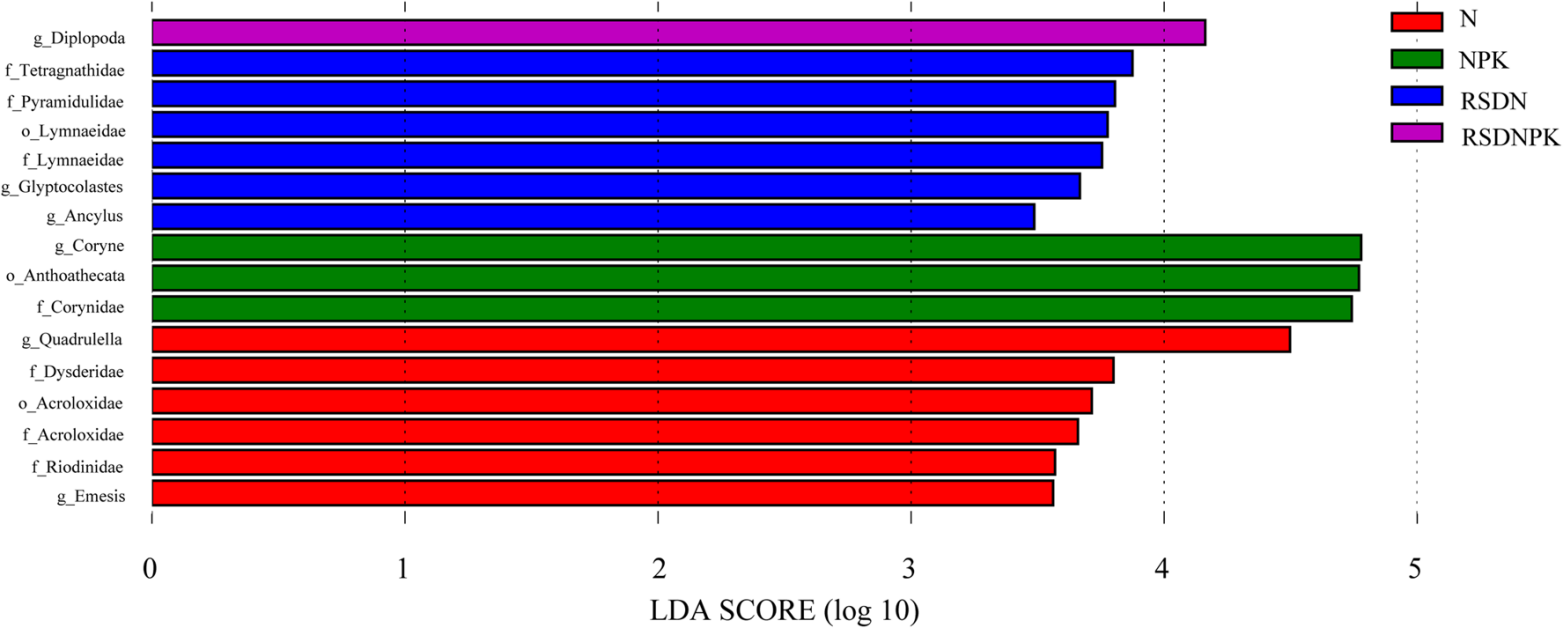
LEfSe analysis of soil faunal taxa in different fertilization treatments. The LDA score represents the different sizes among fertilizer treatments with a threshold value of 2. *N* synthetic N fertilizer, *NPK* synthetic fertilizer: nitrogen, phosphorus and potassium, *RSDN* crop residues returned with nitrogen, *RSDNPK* crop residues returned with nitrogen, phosphorus and potassium.

To further understand the effects of five different kinds of fertilization treatments on the soil faunal taxa, LEfSe analysis (Fig. 2) was used to identify biomarkers in different fertilization treatments. Significantly different abundant taxa were identified in N, including *Quadrulella*, *Emesis*, Riodinidae, Acroloxidae and Dysderidae; Anthoathecata, Coryne, and Corynidae presented the highest LDA score in NPK; *Glyptocolastes*, *Ancylus*, Lymnaeidae, Tetragnathidae and Pyramidulidae were present in high proportions in RSDN; and *Diplopoda* was the biomarker of RSDNPK.

Compared with the N treatment, the relative abundances of Glyptocolastes, *Ancylus*, Tetragnathidae, Lymnaeidae, and Pyramidulidae were increased by crop straw return (RSDN). The relative abundances of Anthoathecata, Coryne, and Corynidae were increased by phosphate fertilizer addition (NPK). In addition, the simultaneous application of straw and phosphate fertilizer (RSDNPK) increased the relative abundance of *Diplopoda*. The results verified that the soil faunal community was relatively sensitive to straw return and phosphate fertilizer addition.

### The impact of straw return and phosphate addition on soil faunal diversity

**Figure 3:**
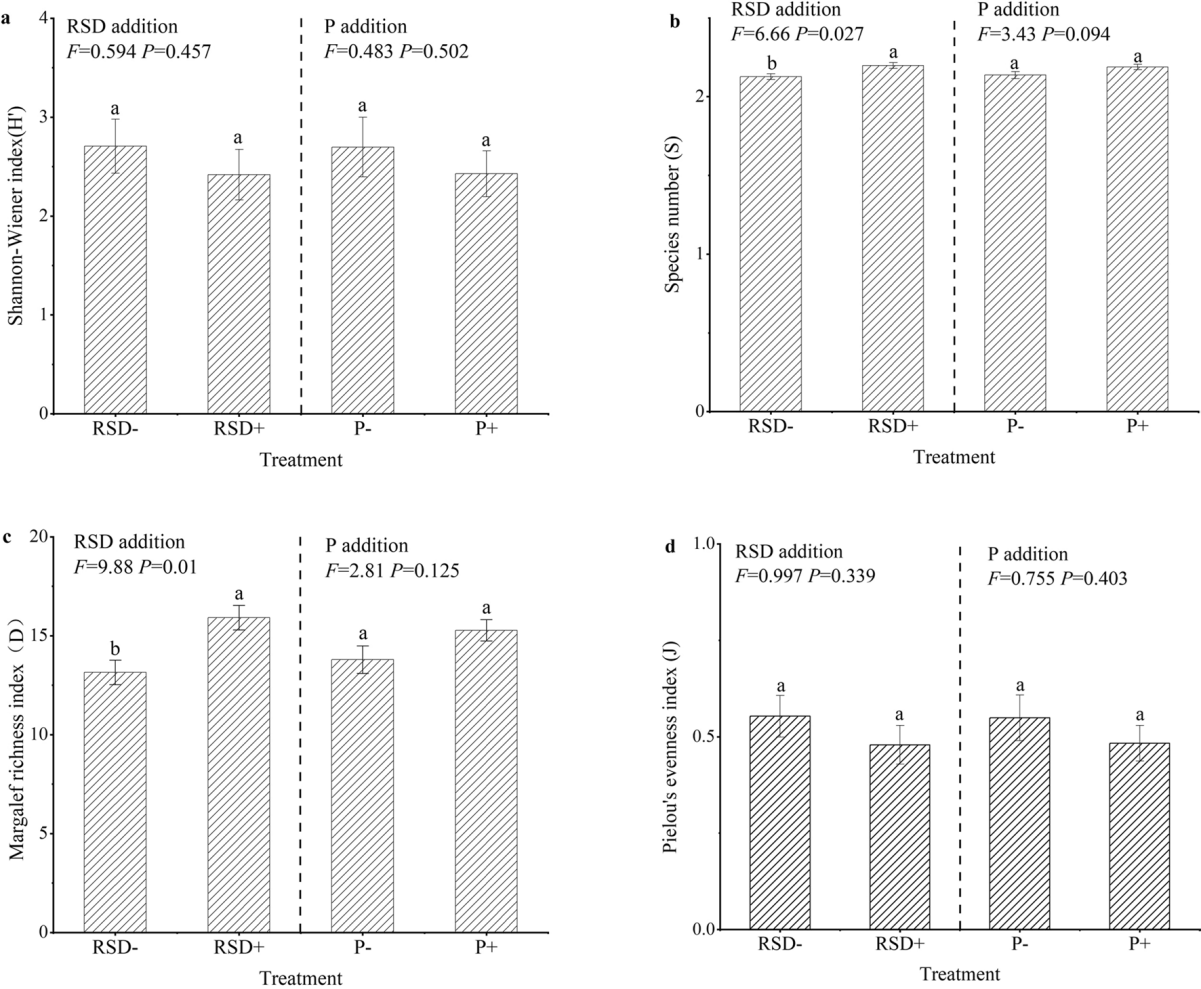
Effects of straw return (RSD) and phosphate fertilizer (P) on the Shannon–Wiener index (H′) (**a**), species number (S) (**b**), Margalef richness index (D) (**c**) and Pielou evenness index (J) (**d**) of the soil faunal community (mean ± standard error). The species number (S) was transformed using log (x). Treatments with different letters (a and b) are significantly different (Two-way ANOVA, *P* < 0.05; n = 3). RSD+ (the treatments with crop straw return): RSDN, RSDNP and RSDNPK; RSD− (the treatments without crop straw return): N and NPK; P+ (the treatments with phosphorus fertilizer): NPK, RSDNP and RSDNPK; P− (the treatments without phosphorus fertilizer): N and RSDN.

Two-way analysis of variance demonstrated that the species number (S) (Fig. 3b;* F* = 6.66; *P* = 0.027) and Margalef richness index (D) (Fig. 3c; *F* = 9.88; *P* = 0.01) were significantly positively affected by straw return, while there was no significant difference in the Shannon–Wiener index (H') (Fig. 3a;* F* = 0.594; *P* = 0.457) and Pielou evenness index (J) (Fig. 3d; *F* = 0.997; *P* = 0.339) between the fertilization regimes. In addition, phosphorus fertilizer had no significant effect on the four indices (Fig. 3a–d; *P* > 0.05).

**Figure 4:**
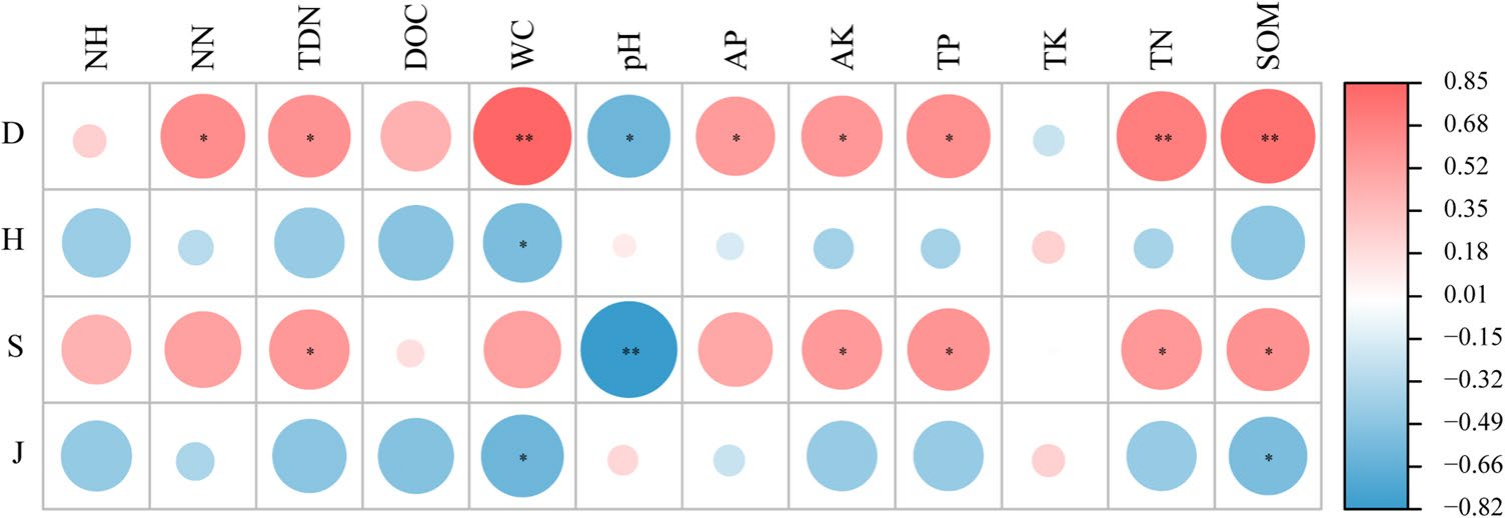
Pearson correlation coefficients between soil faunal diversity and soil factors. The species number (S) was transformed using log (x). *H* Shannon–Wiener index, *D* Margalef richness index, *S* species number, *J* Pielou evenness index, *SOM* soil organic matter, *TN* total nitrogen, *TP* total phosphorus, *TK* total potassium, *AP* available phosphorus, *AK* available potassium, *TDN* dissolved total nitrogen, *DOC* dissolved organic carbon, *NH* ammonium nitrogen, *NN* nitrate nitrogen, *WC* water content. Red represents a positive correlation; blue represents a negative correlation; the depth of color indicates the strength of the correlation. *P < 0.05, **P < 0.01.

The relationship between soil properties and soil faunal diversity was analyzed by Pearson correlation coefficients (Fig. 4). The Margalef richness index (D) was positively correlated with soil NO_3_^–^N, TDN, AP, AK, and TP (*P* < 0.05) and extremely positively correlated with soil WC, SOM and TN (*P* < 0.01), whereas it had a significantly negative correlation with soil pH (*P* < 0.05). Species number (S) had a positive correlation with soil TDN, AK, TP, TN, and SOM (*P* < 0.05) and a negative correlation with soil pH (*P* < 0.01). The Shannon–Wiener index (H') had a negative correlation only with soil WC (*P* < 0.05), and the Pielou evenness index (J) had a negative correlation with soil WC and SOM (*P* < 0.05). These results showed that soil factors such as soil NO_3_^–^N, TP, AP, TDN, TN and SOM had a close relationship with the soil faunal diversity.

### Effect of soil properties on the main soil faunal taxa

**Figure 5:**
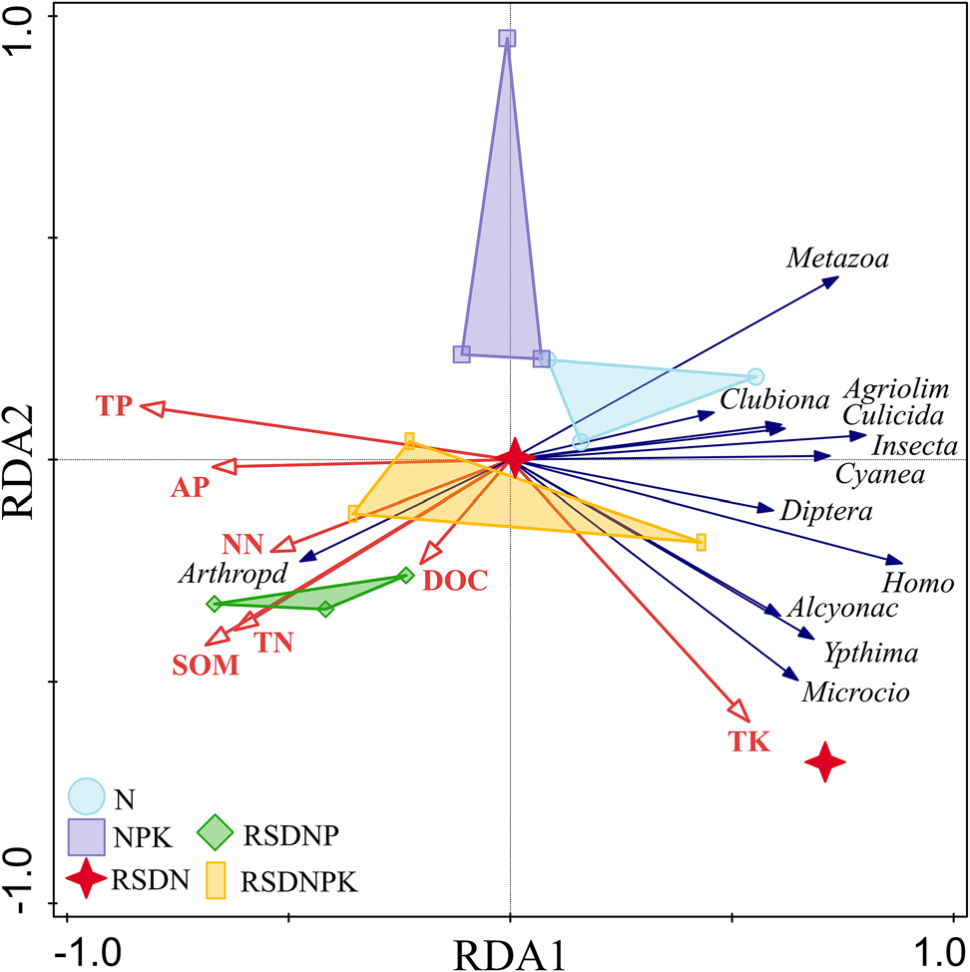
Redundancy analysis of the top 40 abundant taxa of soil fauna with selected soil physical and chemical properties. Arthropd: p_Arthropoda; Insecta: c_Insecta; Alcyonac: o_Alcyonacea; Agriolim: f_Agriolimacidae; Culicida: f_Culicidae; Microcio: f_Microcionidae; *Coenobit*: g*_Coenobita*; *Clubiona*: g_*Clubiona*; *Diptera*: g*_Diptera*; *Cyanea*: g_*Cyanea*; *Ypthima*: g*_Ypthima*; *Homo*: g_*Homo*. *N* synthetic N fertilizer, *NPK* synthetic fertilizer: nitrogen, phosphorus and potassium, *RSDN* crop residues returned with nitrogen, *RSDNP* crop residues returned with nitrogen and phosphorus, *RSDNPK* crop residues returned with nitrogen, phosphorus and potassium, *SOM* soil organic matter, *TN* total nitrogen, *TP* total phosphorus, *TK* total potassium, *AP* available phosphorus, *DOC* dissolved organic carbon, *NN* nitrate nitrogen.

RDA (Fig. 5) was performed to correlate the relative abundance of the soil fauna with the fertilizer treatments to define the major environmental variables that impacted the soil faunal community. The Monte Carlo test illustrated that all of the axis explanatory variables were significant (*F* = 1.5, *P* = 0.004). On the basis of the forward-selection option, all of the environmental variables accounted for 55.9% of the variation in the soil faunal community among the samples. The first canonical axis was mainly determined by soil TP and AP and explained 19.00% of the total variation. The second canonical axis included soil SOM and TK and explained 10.00% of the variation. Furthermore, soil TP (*P* = 0.002) was the major soil environmental factor that influenced the soil faunal community. The abundances of Culicidae, Agriolimacidae, Insecta, Crabronidae, Alcyonacea, Microcionidae, *Diptera*, *Ypthima*, *Clubiona*, *Cyanea*, and *Coenobita* presented negative correlations with soil NO_3_^–^N, DOC, SOM, TN, AP and TP, and they had strong positive correlations with soil TK. The Arthropoda was positively correlated with soil factors (NO_3_^–^N, DOC, SOM, TN, AP and TP). As decomposers, the major soil fauna exhibited sensitive responses to changes in soil fertility.

**Figure 6:**
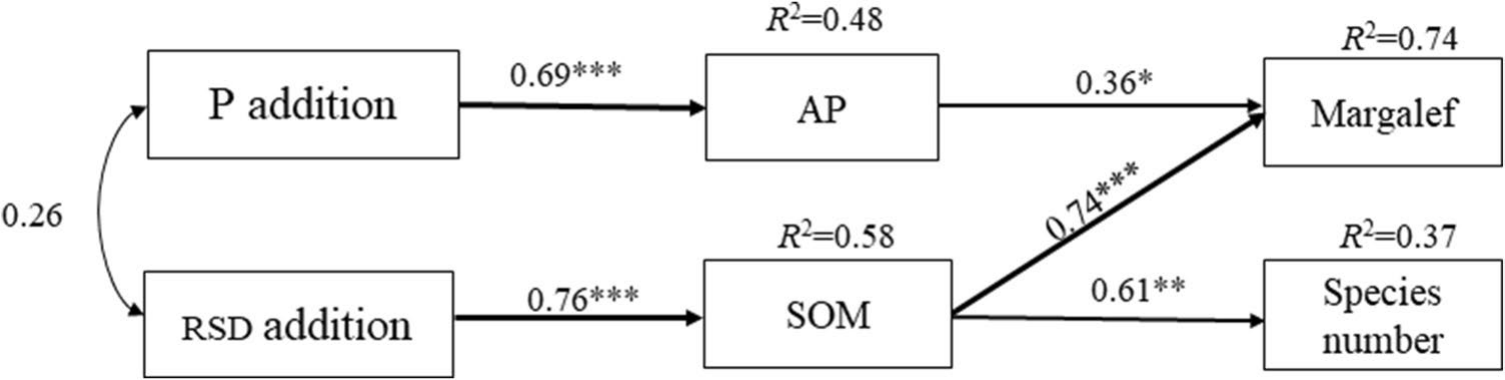
Structural equation model. Black arrows indicate a positive relationship; the number on the arrow is the normalized path coefficient. The *R*^2^ value represents the ratio of variation to the other variables explained, *P < 0.05, **P < 0.01, ***P < 0.001. *SOM* soil organic matter, *AP* available phosphorus.

To further explore the influence of fertility regimes on soil faunal diversity, a structural equation model was established (Fig. 6, *χ*^2^ = 9, CMIN/DF = 0.976, *P* = 0.458, GFI = 0.840, CFI = 1.000, RMSEA = 0.000). The model indicated that the effects of straw return and phosphate fertilizer on soil faunal diversity were significant; the application of phosphate fertilizer to purple soil increased the content of available phosphorus (0.69, *P* < 0.001) and then directly affected the Margalef richness index of the soil fauna (0.74, *P* < 0.05). Straw return increased the soil organic matter content (0.76, *P* < 0.001) and enhanced the soil faunal Margalef richness index (0.74, *P* < 0.001) and species number (0.61, *P* < 0.01).

## Discussion

### Comparison of molecular and morphological techniques in identifying soil nematodes

By exploring the differences between traditional morphological and molecular biological methods in identifying the soil nematode community, we found that the traditional morphological method generated a relatively low number of nematodes. It could be partly explained by identification methods and the artificial bias. Nematodes need water films to survive and move around in the soil^6^. The number of living nematode decreased when the soil samples were collected at a situation of less rainfall, but the genetic information of soil nematode could be detected through the molecular technique. And the COI genetic method generated a larger population of nematode communities and some of them were not recognized by the traditional morphological method (such as *Xiphinema*, *Thelandros* and some parasitic nematodes). In this respect, nematode taxa were more comprehensively detected by the molecular method. However, large proportions of nematode taxa could not be identified to the family or genus level by the molecular technique. And some COI sequences acquired from soil eDNA were microbial (fungi, plants, etc.), a result consistent with Yang et al.^40^ and Horton et al.^41^ The reasons for this may range from DNA extraction to a bias in PCR amplification^42^. The accuracy of sequencing, incomplete databases or primers selection also restricts species identification^43^. In practical analysis, genus-level annotation information is not available for all feature sequences because of incomplete soil faunal databases. Therefore, there is a need to add new and validated reference sequences to the nematode databases for metabarcoding studies^44^. When more taxa are morphologically consistent with the corresponding reference sequences, the molecular biological method for identifying the soil nematode community will be improved. The selected primers determine the accuracy and taxonomic breadth of acquired datasets. It is likely necessary to develop degenerate primers targeting the COI gene to include a broader range of soil nematodes^41^. Despite both the morphological and molecular methods had limitations in characterizing the soil nematode community, the results indicated that the two methods could complement each other to identify soil nematode community.

### Effects of different fertilization on the soil faunal community and diversity

Fertilization affects the soil faunal community, and organic fertilizer promotes soil faunal community diversity^45^. The results of our study confirmed that different fertilization caused changes in the soil faunal community (Adonis test, *R*^2^ = 0.43, *P* = 0.011). Straw return significantly increased soil TN, SOM, TDN and NO_3_^–^N (Table 1), and these altered soil factors exhibited a significant correlation with the soil faunal diversity (Fig. 4). Culicidae, Agriolimacidae, Insecta, Crabronidae, Alcyonacea, Microcionidae, *Diptera*, *Ypthima*, *Clubiona*, *Cyanea*, and *Coenobita* were the dominant taxa in the purple soil under the straw return treatments (Fig. 5). In addition, the return of straw to the field increased the relative abundances of Glyptocolastes, *Ancylus*, Tetragnathidae, Lymnaeidae, and Pyramidulidae (Fig. 2), which showed that these soil faunal taxa were relatively sensitive to straw addition.

Significant effect of straw return on the abundances of earthworms and Collembola and the Shannon–Wiener's index of the soil invertebrates was reported previously^25^. The return of straw to the field increased the contents of nitrogen, phosphorus, potassium and other elements in the soil^19^, providing nutrients for microbes and soil fauna. In addition, straw return can further improve the soil water content, reduce the soil bulk density, increase soil permeability, and provide a variety of habitats^46^. In summary, straw return provided a beneficial environment for the survival of soil fauna.

Phosphorus is a main limiting element in the studied purple soil^23^. Exogenous phosphorus application can improve soil phosphorus availability and the soil nutrient balance to promote soil faunal community diversity^47,48^. The responses of different soil fauna to phosphorus addition are highly heterogeneous in different ecosystems. In the process of litter decomposition in subtropical forests, the diversity and richness of soil arthropods increased through the coaddition of N and P^47^. Phosphate fertilizer was also reported to increase the abundance of Collembola but decreased predatory nematodes in a phosphorus-limited woodland^2^. In this study, phosphate fertilization significantly changed soil AP and TP (Table 1). Meanwhile, the relative abundances of Riodinidae, Dysderidae, *Quadrulella*, Acroloxidae, and *Emesis* decreased in the P addition treatments, while the relative abundances of Anthoathecata, Coryne and Corynidae increased (Fig. 2). Pearson correlation analysis showed significant correlations between soil faunal diversity (Margalef diversity index and species number) and soil TP. The RDA (Fig. 5) further indicated that soil TP had a beneficial effect on soil Arthropoda. This was consistent with a study in forest regions^47^.

## Conclusions

This study investigated the community and diversity of soil fauna in response to crop straw combined with different synthetic fertilizers in upland purple soil using the conserved COI gene fragment in eukaryotic cells as a molecular marker. The results of our study showed that there were significant differences in the community composition of the soil fauna under different fertilization treatments. Straw return significantly increased soil SOM and TN. Soil SOM and AP were the main fertility factors affecting the soil faunal community and diversity. The soil faunal Margalef richness index (D) and species number (S) increased as a result of increases in the soil organic matter and available phosphorus contents. Although both the morphological and molecular methods remained limited in the detection of soil nematodes, which required time to improve. The combination of them was still useful to confirm the results of taxonomical identification. The results imply that crop straw return improves the soil fertility and diversity of soil fauna in upland purple soil. The study provides an important scientific basis and theoretical reference for maintaining agricultural sustainability.

## Materials and methods

### Site description

The study site is located at the Yanting Agro-ecological Experimental Station of Purple Soil (N 31°16′, E 105°28′) of the Chinese Academy of Sciences (CAS). It is located at an altitude of 400–600 m in the middle of the Sichuan Basin, southwestern China. The climate at the site is a moderate subtropical monsoon climate, with a mean annual temperature of 16.3 °C and a mean annual precipitation of 756 mm (2002–2019). The annual precipitation in this region is concentrated in the period from June to October when maize is planted. The vegetation consists mainly of artificial alder (*Alder cremastogyne*), cypress (*Cypressus funebris*) mixed forest, and the main crops are rice (*Oryza sativa*), corn (*Zea mays* L.), wheat (*Triticum aestivum*), sweet potato (*Ipomoea, batatas* Lam.), and rape (*Brassica campestris* L.). The soil used in this experiment is classified as a Pup-Orthic-Entisol in the Chinese Soil Taxonomy, and has a clay loam texture^49^. The soil properties are as follows: loose soil, good permeability, soil thickness of 20–60 cm, soil pH of 8.2–8.3, calcium carbonate content higher than 6%, approximately 10 g/kg soil organic matter, low nitrogen and phosphorus contents, serious zinc and boron deficiencies, shallow soil, and poor water retention and drought resistance^50^.

### Experimental design

The experiment was initiated in 2002. The experiment followed a randomized block design consisting of fifteen plots with five fertilization treatments. Each treatment consisted of three replicate plots (24 m^2^ each, slope 6.5°). The five fertilization treatments were as follows: RSDN (synthetic N nitrogen: 200 kg N ha^−1^ y^−1^ plus crop straw returned: average 12.25 t ha^−1^ y^−1^, which provided 80 kg N ha^−1^ y^−1^), RSDNP (synthetic fertilizer, nitrogen: 200 kg N ha^−1^ y^−1^, phosphorus: 180 kg P_2_O_5_ ha^−1^ y^−1^ plus crop straw returned: average 12.25 t ha^−1^ y^−1^, which provided 80 kg N ha^−1^ y^−1^) and RSDNPK (synthetic fertilizer, nitrogen: 200 kg N ha^−1^ y^−1^, phosphorus: 180 kg P_2_O_5_ ha^−1^ y^−1^, potassium: 72 kg K_2_O ha^−1^ y^−1^ plus crop straw returned: average 12.25 t ha^−1^ y^−1^, which provided 80 kg N ha^−1^ y^−1^) and treatments without straw, i.e., N (synthetic N fertilizer: 280 kg N ha^−1^ y^−1^), NPK (synthetic fertilizer, nitrogen: 280 kg N ha^−1^ y^−1^, phosphorus: 180 kg P_2_O_5_ ha^−1^ y^−1^, and potassium: 72 kg K_2_O ha^−1^ y^−1^), The chemical N, P, and K fertilizers were ammonium bicarbonate (2002–2017, N 17%)/urea (since September 2017, N 46.3%), superphosphate (P_2_O_5_ 12%), and potassium chloride (K_2_O 60%), respectively. The fertilizer application details were shown in Table 2.

**Table 2:** Details of the fertilizer application in the experiment.

Rotation system	Fertilizer type	Treatment				
		N	NPK	RSDN	RSDNP	RSDNPK
Wheat season (October to May)	Chemical N (kg ha^−1^)	130	130	80	80	80
	Maize straw N (kg ha^−1^)	0	0	50	50	50
	P_2_O_5_ (kg ha^−1^)	0	90	0	90	90
	K_2_O (kg ha^−1^)	0	36	0	0	36
Maize season (May to September)	Chemical N (kg ha^−1^)	150	150	120	120	120
	Wheat Straw N (kg ha^−1^)	0	0	30	30	30
	P_2_O_5_ (kg ha^−1^)	0	90	0	90	90
	K_2_O (kg ha^−1^)	0	36	0	0	36
Total amount of nitrogen applied per year (kg ha^−1^)		280	280	280	280	280

*N* synthetic N fertilizer, *NPK* synthetic fertilizer: nitrogen, phosphorus and potassium, *RSDN* crop residues returned with nitrogen, *RSDNP* crop residues returned with nitrogen and phosphorus and *RSDNPK* crop residues returned with nitrogen, phosphorus and potassium.

In each plot, the cropping system was a wheat–maize rotation for all treatments each year during the whole 18-year experiment. Wheat–maize rotation is a typical intensive agricultural technique in the Sichuan Basin of the upper Yangtze River, China, which is in accordance with the local farming system^50^. Winter wheat (*Triticum aestivum* L*.*) was planted by direct sowing from late October to May of the following year, and summer maize (*Zea mays* L*.*) was planted in planting holes from May to September^51^. The returned straws were harvested from a large adjacent field, which was managed in accordance with NPK treatment and the nutrients content of straw were measured. Maize straw [TN (total organic nitrogen) 0.8%; TOC (total organic carbon) 44%] was returned to the soil before the wheat was planted, and wheat straw (TN 0.5%; TOC 43%) was returned to the soil before the maize was planted. The maize and wheat straws were chopped into small pieces (approximately 5 cm long) and then applied to the field according to the mulching pattern. All chemical fertilizers and organic materials were manually applied to the soil at a depth of 10 cm and once as basal fertilization on the day of sowing^51^.

### Research involving plants

Plant material and soil were collected from an experimental station, permissions were obtained from Yanting Agro-ecological Experimental Station of Purple Soil of the Chinese Academy of Sciences (CAS).

Experimental research on plants and soil, including the collection of plant material and soil comply with relevant institutional, national, and international guidelines and legislation.

## Soil sampling and analysis

### Soil sampling

Surface soil (0–15 cm, 1 kg) samples were collected with trowels for each treatment during the fallow period of the wheat–maize rotation in May 2019. Five soil cubes were randomly collected from each fertilization plot and mixed together to generate one composite sample (n = 15). All samples were immediately transported to the laboratory and passed through a 2 mm sieve after removing roots and stones. One subsample was air-dried for soil property analysis, another subsample was stored at 4 °C for nematode morphological identification, and the remaining subsample was immediately stored at − 80 °C for molecular analysis.

### Analyses of soil properties

Dissolved total nitrogen (TDN), ammonium nitrogen (NH_4_^+^-N), and nitrate nitrogen (NO_3_^–^N) were analyzed by an Auto Analyzer-AA3 (SEAL, Germany). Total nitrogen (TN) was measured with an elemental analyzer (Vario MICRO cube analyzer, Elementar, Germany). Total phosphorus (TP) was determined by the molybdenum blue colorimetric method following digestion with perchloric acid (HClO_4_)^52^_._ Dissolved organic carbon (DOC) was extracted with 0.5 M K_2_SO_4_ and analyzed using a TOC-5000 analyzer (Shimadzu, Kyoto, Japan). Soil organic matter (SOM) was analyzed by the dichromate oxidation spectrophotometric method^53^. Soil pH was measured in soil–water suspensions (1:2.5 = W:V). The soil water content (WC) was measured after oven–drying for 24 h at 105 °C^54^. Soil total potassium (TK) was determined by H_2_SO_4_/H_2_O_2_ digestion and atomic absorption spectrophotometry. Available potassium (AK) was determined based on 1 mol/L ammonium acetate (NH_4_OAc)-extractable K^55^. Olsen-P (available phosphorus) was extracted with 0.5 mol/L sodium bicarbonate (NaHCO_3_) at pH 8.5^56^.

### Identification of soil nematodes by the morphological method

Nematodes were extracted from 50 g of moist soil using the Baermann funnel method for 48 h^57^. After being preserved in 4% formalin solution, the total number of soil nematodes was counted using an inverted composite microscope (Olympus SZX16, Japan). Nematode abundance was expressed as the number of nematodes per 100 g of dry soil. After counting the total nematode abundance in each sample, one hundred individuals were randomly selected and identified to the genus level at 400 × magnification^51^.

### DNA extraction and COI gene amplicon sequencing

Total genomic DNA samples were extracted using Fast DNA SPIN extraction kits (MP Biomedicals, Santa Ana, CA, USA) following the manufacturer’s instructions and were stored at − 20 °C prior to further analysis. The quantity and quality of extracted DNA were measured using a NanoDrop ND-1000 spectrophotometer (Thermo Fisher Scientific, Waltham, MA, USA) and agarose gel electrophoresis, respectively. PCR amplification of the COI gene was performed using the forward primer *COIintF* (5′-GGWACWGGWTGAACWGTWTAYCCYCC-3′) and the reverse primer *COIjgHCO2198* (5′-TANACYTCNGGRTGNCCRAARAAYCA-3′)^58^. Sample-specific 7-bp barcodes were incorporated into the primers for multiplex sequencing. The PCR mixture contained 5 μl of Q5 reaction buffer (5 ×), 5 μl of Q5 High-Fidelity GC buffer (5 ×), 0.25 μl of Q5 High-Fidelity DNA Polymerase (5 U/μl), 2 μl (2.5 mM) of dNTPs, 1 μl (10 µM) of each forward and reverse primer, 2 μl of the DNA template, and 8.75 μl of ddH_2_O. Thermal cycling consisted of initial denaturation at 98 °C for 2 min, followed by 25 cycles consisting of denaturation at 98 °C for 15 s, annealing at 55 °C for 30 s, and extension at 72 °C for 30 s, with a final extension of 5 min at 72 °C^59^. PCR amplicons were purified with Agencourt AM Pure Beads (Beckman Coulter, Indianapolis, IN) and quantified using the PicoGreen dsDNA Assay Kit (Invitrogen, Carlsbad, CA, USA). After the individual quantification step, amplicons were pooled in equal amounts, and paired-end 2 × 300 bp sequencing was performed using the Illumina MiSeq platform with MiSeq Reagent Kit v3 at Shanghai Personal Biotechnology Co., Ltd. (Shanghai, China).

### Sequence analysis

The Quantitative Insights into Microbial Ecology (QIIME, v1.8.0) pipeline was employed to process the sequencing data, as previously described^60^. Briefly, raw sequencing reads with exact matches to the barcodes were assigned to respective samples and identified as valid sequences. Low-quality sequences were filtered through the following criteria: sequences that had a length < 150 bp, sequences that had average Phred scores < 20, sequences that contained ambiguous bases, and sequences that contained mononucleotide repeats > 8 bp. Paired-end reads were assembled using FLASH^61^. After chimera detection, the remaining high-quality sequences were clustered into operational taxonomic units (OTUs) at 97% sequence identity by UCLUST^62^. A representative sequence was selected from each OTU using default parameters. OTU taxonomic classification was conducted by BLAST searching the representative sequences against the NCBI NT Database using the best hit^63^. An OTU table was further generated to record the abundance of each OTU in each sample and the taxonomy of these OTUs. OTUs containing less than 0.001% of total sequences across all samples were discarded. To minimize the difference in sequencing depth across samples, an averaged, rounded rarefied OTU table was generated by averaging 100 evenly resampled OTU subsets under 90% of the minimum sequencing depth for further analysis. Environmental DNA sequences have been deposited in the NCBI Sequence Read Archive (Project Accession: PRJNA879992).

### Statistical analysis and data visualization

One-way analysis of variance (ANOVA) was used to test the effects of different fertilization treatments on soil properties. ANOVA was also used to test the soil nematode abundance and diversity (identified by the morphological method) under different treatments with SPSS 22.0 software (SPSS IBM Inc., USA), followed by the least significant difference test (LSD, *P* < 0.05). The difference in the total soil faunal community among the five fertilization treatments (n = 15) was tested by the Adonis test, which was verified by the R 4.0.2 ‘vegan’ package^64^. A heatmap (*n* = 15) was plotted using heatmap tools in the Genescloud platform (https://www.genescloud.cn). The tool was developed from the ‘pheatmap’ package (V 1.0.8), which was slightly modified to improve the layout style. The top 40 abundant soil faunal taxa among the five fertilization treatments were normalized by z scores and transformed using log10. The clustering method was complete (default), and the list of distances was Euclidean (default). Biomarkers for soil faunal taxa between the five fertilization regimes were found by LEfSe analysis, which was conducted using a free online platform for data analysis (http://www.cloud.biomicroclass.com/CloudPlatform/home). The alpha value of the Kruskal–Wallis test was set to 0.05, and the LDA threshold was set to 2.

Two-way analysis of variance (ANOVA) was used to test the effects of crop straw return and P addition on soil faunal diversity. The characteristics of the soil faunal community were described using the following indices, which were calculated using Excel 2016 (Microsoft Inc., USA) and R 4.0.2: (1) Shannon–Wiener index (H′)^65^, H′ = −$\sum\nolimits_{i = 1}^{S} {P_{i} \;ln\;P_{i} }$, where Pi is the proportion of the community represented by OTUs and S is the number of OTUs; (2) Margalef richness index (D)^66^, which is an indicator of species richness, D = (S − 1)/ln N, where N is the total number of individuals of all taxa observed and S is the number of OTUs. (3) Pielou evenness index (J)^67^, which represents the degree of uniformity, J = H′/lnS. The bar graph (Fig. 3) was drawn using Origin 2019 (Origin Lab Inc., USA). (4) species number (S), which is the number of species^68^. The correlations between the soil faunal diversity (D, J, S, H′) and soil properties (NO_3_^–^N, NH_4_^+^-N, TP, AP, TDN, TN, AK, TK, DOC, WC, pH and SOM) were analyzed by Pearson correlation coefficients. The species number (S) was transformed using log (x). The analysis was performed by the gene cloud tools, a free online platform for data analysis (https://www.genescloud.cn).

Redundancy analysis (RDA) was used to analyze the relationships between the soil faunal relative abundance and soil fertility variables. The 40 most abundant taxa were selected, and their relative abundance was transformed using log (x + 1) and center transformation. Soil environmental variables were selected by a forward selection procedure to obtain the best explained variables of the soil faunal distribution and were used in the analysis. The statistical significance of the RDA was assessed by the Monte Carlo permutation test (*P* < 0.05) using CANOCO 5 (Microcomputer Power Inc., USA).

We constructed a structural equation model (SEM) with the specification of a conceptual model of hypothetical relationships, assuming that the SOM and P addition alter soil SOM, TP, AP, TN, TDN, WC, and NO_3_^–^N, which, in turn, affect the soil faunal Margalef richness index (D) and species number (S). Amos 23 (SPSS, IBM Inc., USA) software was used for SEM analysis, and the data were fitted to the models using the ‘robust’ maximum likelihood estimation procedures. The model was assessed based on the *χ*^2^ value (*P* > 0.05, CMIN/df < 2), goodness of fit (GFI) (values > 0.8 and < 1), the comparative fit index (CFI) (values > 0.9), and the root square mean error of approximation (RMSEA) (values < 0.05)^69^. Then, by progressively removing nonsignificant relationships between the remaining observed variables, we inferred the final model (Fig. 6). The *R*^2^ values were obtained for each dependent matrix, representing the proportion of total variance explained by the model^70,71^. All significant differences were set at a level of *P* < 0.05.

## Supplementary Information

Supplementary Table S1.

Supplementary Table S2.

## Data Availability

The datasets used and/or analyzed during the current study are available from the corresponding author on reasonable request.
