# Supplementary material for: Diversity of soil faunal community as influenced by crop straw combined with different synthetic fertilizers in upland purple soil
Source: Sci Rep. 2022 Nov 11;12:19306. doi: 10.1038/s41598-022-23883-6 (PMC9652334; doi:10.1038/s41598-022-23883-6)
Supplement: Supplementary file 1 — Supplementary Table S1. [file 41598_2022_23883_MOESM1_ESM.docx]

**Diversity of soil faunal community as influenced by continuous crop straw combined with different synthetic fertilizers in upland purple soil**

Xiuhong Xie^1, 2, 3^, Xuefeng Wang^4^, Zhixin Dong^1, 2^ & Bo Zhu ^1, 2^

^1^ Key Laboratory of Mountain Surface Processes and Ecological Regulation, Institute of Mountain Hazards and Environment, Chinese Academy of Sciences, Chengdu, 610041, China

^2^ Institute of Mountain Hazards and Environment, Chinese Academy of Sciences, Chengdu, 610041, China

^3^ University of Chinese Academy of Sciences, Beijing, 100049, China

^4^ Jilin Agricultural University, Changchun, 130118, China

**Supplementary Table 1 Soil nematode community by morphological methods**

| **Trophic groups** | **Class** | **Order** | **Family** | **Genus** | **Fertilization regimes** | | | | |
| --- | --- | --- | --- | --- | --- | --- | --- | --- | --- |
|  |  |  |  |  | **N** | **NPK** | **RSDN** | **RSDNP** | **RSDNPK** |
| PP | Chromadorea | Rhabditida | Tylenchidae | *Coslenchus* | 0 | 0 | 4 | 1 | 0 |
|  |  |  |  | *Aglenchus* | 0 | 0 | 0 | 0 | 3 |
|  |  |  |  | *Tylenchus* | 1 | 2 | 0 | 2 | 0 |
|  |  |  |  | *Psilenchus* | 1 | 0 | 1 | 1 | 0 |
|  |  |  | Dolichodoridae | *Pratylenchus* | 1 | 1 | 1 | 0 | 1 |
|  |  |  |  | *Dolichorhynchus* | 0 | 0 | 0 | 0 | 1 |
|  |  |  |  | *Telotylenchus* | 1 | 0 | 1 | 1 | 0 |
|  |  |  |  | *Tylenchorhynchus* | 0 | 1 | 0 | 2 | 1 |
|  |  |  | Hoplolaimidae | *Helicotylenchus* | 4 | 0 | 1 | 0 | 0 |
|  |  |  |  | *Rotylenchus* | 1 | 0 | 0 | 2 | 0 |
|  |  |  | Criconematidae | *Macroposthonia* | 1 | 0 | 1 | 0 | 0 |
|  |  |  | Aphelenchoididae | *Aphelenchoides* | 0 | 5 | 2 | 2 | 1 |
|  |  |  | Anguinidae | *Ditylenchus* | 0 | 1 | 1 | 1 | 0 |
|  | Enoplea | Triplonchida | Trichodoridae | *Paratrichodorus* | 1 | 0 | 2 | 0 | 0 |
| FF | Chromadorea | Rhabditida | Aphelenchidae | *Aphelenchus* | 1 | 1 | 1 | 1 | 3 |
|  |  |  |  | *Paraphelenchus* | 0 | 1 | 0 | 2 | 1 |
|  |  |  |  | *Filenchus* | 0 | 1 | 3 | 0 | 0 |
|  | Enoplea | Triplonchida | Diphtherophoridae | *Tylolaimophorus* | 2 | 0 | 0 | 0 | 0 |
|  |  | Dorylaimida | Leptonchidae | *Tylencholaimus* | 0 | 0 | 1 | 0 | 1 |
| BF | Chromadorea | Rhabditida | Rhabditidae | *Rhabditidae* | 0 | 0 | 1 | 0 | 0 |
|  |  |  |  | *Mesorhabditis* | 0 | 7 | 8 | 0 | 4 |
|  |  |  | Cephalobidae | *Eucephalobus* | 2 | 1 | 0 | 0 | 0 |
|  |  |  |  | *Heterocephalobus* | 1 | 0 | 1 | 0 | 1 |
|  |  |  |  | *Acrobeles* | 0 | 0 | 0 | 0 | 0 |
|  |  |  |  | *Acrobeloides* | 3 | 8 | 16 | 3 | 3 |
|  |  |  |  | *Cervidellus* | 0 | 1 | 1 | 0 | 0 |
|  |  | Plectida | Plectidae | *Plectus* | 0 | 3 | 3 | 9 | 1 |
|  | Enoplea | Mononchida | Mylonchulidae | *Megaonchulus* | 1 | 0 | 1 | 1 | 1 |
|  |  | Triplonchida | Prismatolaimidae | *Prismatolaimus* | 2 | 1 | 0 | 0 | 0 |
|  | Adenophorea | Desmodorida | Microlaimidae | *Microlaimus* | 0 | 2 | 0 | 0 | 1 |
|  |  | Enoplida | Alaimidae | *Paramphidelus* | 0 | 0 | 0 | 0 | 1 |
| OP | Enoplea | Chromadorida | Achromadoridae | *Achromadora* | 1 | 1 | 1 | 0 | 0 |
|  |  | Dorylaimida | Qudsianematidae | *Thonus* | 2 | 0 | 0 | 1 | 2 |
|  |  |  |  | *Eudorylaimus* | 0 | 2 | 1 | 0 | 0 |
|  |  |  |  | *Epidorylaimus* | 1 | 3 | 0 | 6 | 4 |
|  |  |  |  | *Microdorylaimus* | 1 | 2 | 1 | 0 | 0 |
|  |  |  | Aporcelaimidae | *Aporcelaimellus* | 0 | 2 | 0 | 2 | 1 |
|  |  |  | Thornenematidae | *Mesodorylaimus* | 0 | 1 | 0 | 1 | 0 |

The data in the table is an average (*n* = 3)

PP: plant parasites; BF: bacterivores; FF: fungivores; OP: omnivore-predators
