# Supplementary material for: Diversity of soil faunal community as influenced by crop straw combined with different synthetic fertilizers in upland purple soil
Source: Sci Rep. 2022 Nov 11;12:19306. doi: 10.1038/s41598-022-23883-6 (PMC9652334; doi:10.1038/s41598-022-23883-6)
Supplement: Supplementary file 2 — Supplementary Table S2. [file 41598_2022_23883_MOESM2_ESM.docx]

**Diversity of soil faunal community as influenced by crop straw combined with different synthetic fertilizers in upland purple soil**

Xiuhong Xie^1, 2, 3^, Xuefeng Wang^4^, Zhixin Dong^1, 2^ & Bo Zhu ^1, 2^

^1^ Key Laboratory of Mountain Surface Processes and Ecological Regulation, Institute of Mountain Hazards and Environment, Chinese Academy of Sciences, Chengdu, 610041, China

^2^ Institute of Mountain Hazards and Environment, Chinese Academy of Sciences, Chengdu, 610041, China

^3^ University of Chinese Academy of Sciences, Beijing, 100049, China

^4^ Jilin Agricultural University, Changchun, 130118, China

**Supplementary Table 2 Soil nematode community by the COI barcoding method.**

| **Trophic groups** | **Class** | **Order** | **Family** | **Genus** | **Fertilization regimes** | | | | |
| --- | --- | --- | --- | --- | --- | --- | --- | --- | --- |
|  |  |  |  |  | **N** | **NPK** | **RSDN** | **RSDNP** | **RSDNPK** |
| FF | Chromadorea | Rhabditida | Aphelenchoididae | *Bursaphelenchus* | 1 | 2 | 0 | 0 | 3 |
|  |  |  |  | *Schistonchus* | 58 | 1 | 0 | 0 | 0 |
|  |  |  |  |  | 0 | 0 | 0 | 2 | 0 |
|  |  |  | Neotylenchidae | *Fergusobia* | 0 | 1 | 0 | 19 | 0 |
|  |  |  |  |  | 0 | 0 | 0 | 3 | 2 |
| PP | Chromadorea | Rhabditida | Pharyngodonidae | *Thelandros* | 0 | 23 | 0 | 0 | 0 |
|  | Enoplea | Dorylaimida | Longidoridae | *Xiphinema* | 11 | 0 | 0 | 4 | 3 |
| AP | Chromadorea | Strongylida | Ancylostomatidae | *Necator* | 0 | 0 | 0 | 2 | 0 |
|  |  |  |  | *Angiostrongylus* | 0 | 2 | 0 | 0 | 0 |
|  |  |  |  |  | 0 | 0 | 0 | 0 | 1 |
|  |  |  | Strongylidae | *Cylicostephanus* | 0 | 0 | 0 | 715 | 0 |
| OP | Chromadorea | Rhabditida | Heterakidae | *Meteterakis* | 3 | 0 | 0 | 0 | 0 |
|  | Chromadorea | Strongylida |  |  | 0 | 0 | 33 | 16 | 18 |
|  | Chromadorea | Rhabditida |  |  | 2 | 0 | 9 | 436 | 2 |
|  | Chromadorea |  |  |  | 1 | 7 | 8 | 0 | 11 |

The data in the table is an average (*n* = 3)

PP: plant parasites; AP: Animal parasites; FF: fungivores; OP: omnivore-predators
